# Targeting TEAD in cancer

**DOI:** 10.3389/fonc.2025.1692512

**Published:** 2025-11-19

**Authors:** Rohith Battina, Raneen Rahhal, Anton Wellstein, Anna T. Riegel, Ghada M. Sharif

**Affiliations:** Lombardi Comprehensive Cancer Center, Georgetown University Medical Center, Washington D.C., United States

**Keywords:** TEAD family, YAP (Hippo) signaling, TAZ (Hippo) signaling, TEAD inhibitors, YAP/TAZ TEAD signaling

## Abstract

The Hippo pathway is dysregulated in many cancers, leading to pro-oncogenic effects. The transcription factor TEAD plays a critical role in early development, tissue homeostasis, and cell proliferation, and it binds to the downstream Hippo pathway co-activators YAP and TAZ. Numerous studies have examined the roles of YAP/TAZ and TEAD in cancer, with their activity frequently linked to poor clinical prognosis. This review discusses how targeting TEAD interactions with coregulators—most notably YAP and TAZ—represents a promising therapeutic strategy in oncology. Several pharmacological agents have been developed to disrupt the YAP/TAZ–TEAD complex, and many are currently being evaluated for clinical applicability across diverse cancer types. We review current knowledge on the structure and homology of TEAD, emphasizing the protein–protein interfaces that mediate binding to YAP/TAZ and other cofactors. Advances in understanding the YAP/TAZ–TEAD complex have informed the development of diverse strategies to inhibit downstream transcription of key oncogenic target genes. Finally, we highlight TEAD inhibitors currently in clinical trials, outlining their mechanisms of action, associated adverse effects, and potential impact on the future therapeutic landscape.

## 1 Introduction

The Hippo pathway is a tumor suppressor pathway that regulates cellular proliferation and organ size. Dysregulation of the Hippo pathway and increased YAP/TAZ-TEAD transcription plays an essential role in many human malignancies. It is involved in tumor growth, metastasis, immune evasion, and resistance to therapy. Inhibition of the YAP/TAZ-TEAD transcriptional complex using novel TEAD inhibitors have shown promising results for therapeutic targeting in cancer. Currently, TEAD inhibitors are being clinically investigated in cancers with inactivating upstream mutations in the Hippo pathway leading to dysregulation of cellular proliferation.

## 2 TEAD transcription factor family

TEADs (transcription enhancer with TEA domain) are a family of transcription factors involved in early development, tissue homeostasis, cell growth, and proliferation. The discovery of the TEAD family began in the late 1980s with the purification of TEAD1/TEF1, which was shown to specifically bind to the GT-IIC and Sph motifs of the simian virus 40 (SV40) enhancer in HeLa cells (1, 2). Importantly, TEAD also binds the M-CAT (muscle-specific cytidine-adenosine-thymidine) motif, and has demonstrated its necessity to proper cardiac and skeletal muscle development (3). Further studies revealed that TEADs are evolutionarily conserved transcription factors sharing homology with AbaA and TEC1 in yeast, and Scalloped in Drosophila (4). This high degree of conservation in the consensus sequence within the DNA binding domain, spanning approximately 70 amino acid residues, lead to the TEA domain also being referred to as the ATTS domain (AbaA, TEC1, TEF1, Scalloped) (1, 5–7).

Mammals express genes that encode four members of the TEAD transcription family, denoted TEAD1-4. Despite their homology, expression of these TEADs varies between tissues throughout different stages of development (8). TEAD1 has been well established to be a key player in cardiogenesis, driving the proliferation and differentiation of cardiomyocytes (9, 10). Notably, mice harboring a TEAD1 loss of function mutation, are embryonically lethal due to defects in cardiac development (11). Interestingly, cardiomyocyte deletion of TEAD1 leads to perinatal lethality, while overexpression of TEAD1 in striated muscle cells resulted in cardiac dysfunction further demonstrating the importance of TEAD1 in cardiac development and function (9, 12). Unlike other TEADs, TEAD2 is almost completely absent from adult tissues, and is selectively expressed during embryonic development and can be found at the 2-cell zygotic stage (13, 14). Inactivation of TEAD2 in mice led to an increased risk of exencephaly, demonstrating the importance of TEAD2 for neural development (15). Intriguingly, some groups have found that TEAD2 knockout mice actually produced viable adult offspring, suggesting further studies are required to better understand the requirement of TEADs during development and potential compensatory mechanisms (15). TEAD3 has not been widely investigated, and there are no reported knockout studies to date. However, *in vitro* studies have revealed that TEAD3 is expressed in the labyrinthine region of the placenta and is upregulated during the differentiation of cytotrophoblasts to syncytiotrophoblasts (16). It is found in myoblastic tissue and regulates epidermal proliferation in conjunction with TEAD1 (17, 18). TEAD4 plays a crucial role in embryonic development, specifically in trophectoderm lineage specification which influences the placenta and inner cell mass (19). Inactivation of TEAD4 in mice disrupted pre-implantation and was thus lethal (19). Conversely, ablation of TEAD4 post-implantation, yielded normal mouse embryos (20). All four members of the TEAD family appear to have tissue-specific functions, playing an important role in development.

## 3 The Hippo pathway

Although TEADs have been shown to play an important role in development, they are most notably known for being a binding partner of YAP/TAZ, the downstream transcriptional coactivators of the Hippo signaling pathway. Together, the YAP/TAZ-TEAD complex induces the transcription of genes regulating apoptosis, cell proliferation, and organ size (21–25).

**Figure 1:**
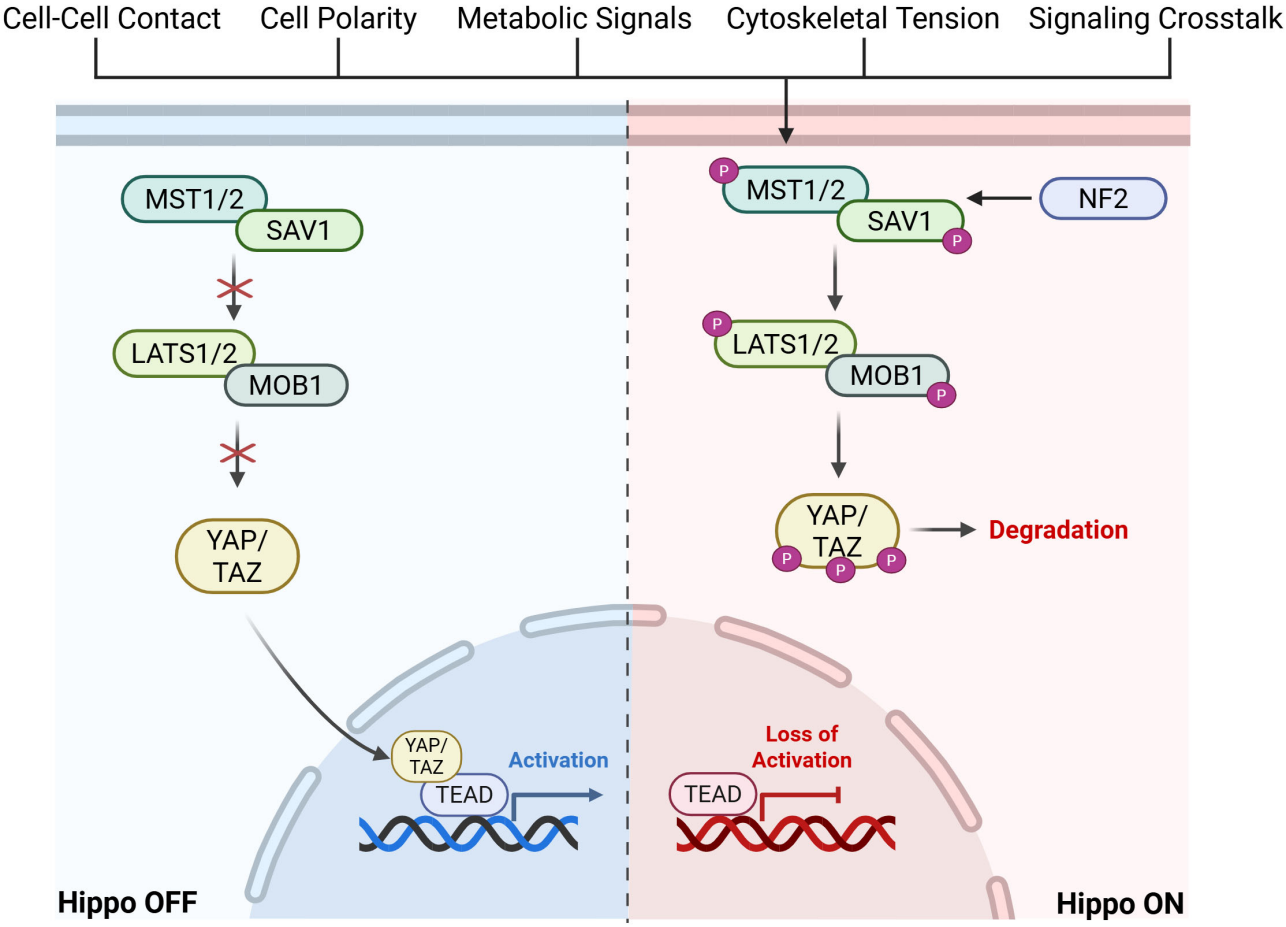
Graphical visualization of Hippo Pathway kinase cascade leading to activation or inactivation of downstream TEAD signaling. Created in BioRender. Battina, R. (2025) https://BioRender.com/1b0g0po.

The Hippo pathway is a highly conserved pathway with core components discovered in, but not limited to, *Drosophila* and mammals (26). It involves a kinase cascade where proteins phosphorylate and activate one another, ultimately regulating the phosphorylation of YAP and TAZ and their interaction with TEADs (**Figure 1**).

In mammalian cells, the Hippo pathway is triggered by upstream factors such as cell-cell contact, cell polarity, metabolic signals, cytoskeletal tension, and GPCR signaling cross-talk. An important upstream factor that leads to activation of the Hippo pathway is Neurofibromatosis type 2 (NF2). NF2 is a mediator of cytoskeletal and membrane-derived signals in response to increased cell-cell contact and it inhibits further cell proliferation by recruiting MST1/2 (27). This upstream activation results in the phosphorylation of MST1/2 (mammalian STE20-like kinase 1/2) (28). MST1/2 and SAV1 (scaffold protein Salvador homologue 1) phosphorylate LATS1/2 (large tumor suppressor kinase 1/2) and MOB1 (scaffold MOBKL1A/B). This activates LATS1/2, allowing it to directly phosphorylate YAP and TAZ. This phosphorylation sequesters YAP/TAZ in the cytoplasm, preventing localization to the nucleus and eventual degradation (27). When the Hippo pathway is inactivated, or “off”, YAP/TAZ is shuttled to the nucleus and forms a complex with TEAD, activating the transcription of a number of genes including those associated with cell proliferation. When the Hippo pathway is dysregulated, there is increased transcriptional activation of downstream targets which have been shown to play a role in metastatic growth and tumor progression in various cancers (29). Importantly, downstream signaling initiated by the Hippo pathway is highly reliant on TEAD, making it an ideal target for therapy.

## 4 Relevance of the YAP/TAZ-TEAD axis in cancer progression

TEADs are key regulators of the Hippo pathway, controlling the transcription of genes involved in cell proliferation and apoptosis. Dysregulation of the Hippo pathway leads to uncontrolled cell growth, a hallmark of cancers, and the expression of oncoproteins promoting invasion and metastasis (30). For example, NF2 mutations have been implicated in multiple cancers including malignant mesothelioma, meningioma, and schwannomas and it was found that YAP/TEAD pathway inhibition blocked growth and survival of these NF2-deficient tumors (31–36). A comprehensive analysis of over 10,000 cancer patient samples from The Cancer Genome Atlas revealed that over 30% of samples carried genetic alterations in core components of the Hippo pathway (29). However, no one gene was altered in more than 10% of patient samples, indicating that the aberrant signaling of the Hippo pathway can be caused by multiple members of the pathway or even through interactions with other signaling pathways (29, 37). For example, PI3K signaling blocks the phosphorylation and activation of upstream Hippo proteins, allowing YAP to localize to the nucleus, and promote the transcription of pro-tumorigenic genes (38). Further pathways, notably the Wnt, TGFb, and EGFR pathways have also been shown to promote dysregulation of the Hippo pathway, promoting tumorigenesis, metastasis, and drug resistance (39).

Most studies examining Hippo signaling in cancer have predominantly focused on YAP/TAZ activity and their effect on tumor initiation and growth. For example, initial studies on the Hippo pathway in the liver demonstrated that high levels of endogenous YAP can lead to hepatomegaly and tumorigenesis (40, 41). Constitutively active TAZ induced breast cancer stem cell properties and tumor formation to further triple negative breast cancer (TNBC) proliferation (42). Interestingly, overexpression of TAZ in low-expressing breast cancer cell lines promoted epithelial-mesenchymal transition (EMT) and led to an increase in migration and invasion (43, 44). An increase in invasion and CSC-like tumorigenic properties was also observed in gastric epithelial cells as a result of YAP/TAZ activation (45, 46). Silencing YAP or TAZ activity *in vitro* has further demonstrated the importance of these oncoproteins to promote tumorigenesis. Knockdown of YAP expression in pancreatic cancer cell lines such as, PANC-1 and BxPC3, led to a reduction in cellular proliferation (47, 48). Additionally, RNAi repression of YAP expression in prostate cancer cell lines reduced proliferation and induced apoptosis (49). Knocking down YAP/TAZ in osteosarcoma cell lines MG-63, HOS, and U2OS reduced cellular proliferation and invasion, while depletion of TAZ in gastric carcinoma cell lines inhibited motility and invasion (50–53). The suppression of YAP/TAZ activity in *in vivo* studies of breast cancer, pancreatic cancer, and osteosarcoma have also demonstrated the contribution of these downstream Hippo proteins on tumorigenesis (51, 54, 55). Furthermore, YAP/TAZ activation can overcome anti-proliferative effects of other inhibitors such as KRAS^G12C^ inhibitors leading to resistance and tumor growth (56). This indicates concurrent treatment with TEAD inhibitors could enhance antitumor activity and prevent acquired resistance to other pathway inhibitors.

In many cancers, increased levels of YAP/TAZ in the nucleus is correlated with poor patient prognosis and an increase in therapeutic resistance (57). In non-small cell lung cancer (NSCLC), increased expression of YAP/TAZ was found to be correlated with worse clinical outcomes, and a shorter overall survival (OS) (58, 59). Inhibition of TEAD also led to increased chemotherapeutic sensitivity in paclitaxel-resistant A549 cells (60). High levels of YAP in small cell lung cancer (SCLC), is not only associated with a shorter OS, but also with radiation and drug resistance (61, 62). Within triple negative breast cancer (TNBC), nuclear expression of YAP/TAZ is strongly associated with and showed poorer clinical outcomes for disease-free survival (DFS) and OS (63–65). Studies stratifying colorectal cancer patients based on YAP activity found YAP activation was highly associated with lower disease-free survival (DFS) and progression-free survival (PFS) (66–68). Immunohistochemistry staining of YAP and TAZ in samples of patients with gastric cancer or hepatocellular carcinoma, also revealed that YAP/TAZ expression was negatively correlated with OS and PFS (69–71). Additionally, tissue microarray analysis of over 17,000 prostate cancer samples showed that elevated YAP protein levels were associated with more advanced tumor staging and earlier biochemical recurrence (72, 73). Given that the majority of YAP/TAZ transcriptional activity occurs through binding with TEAD, this continues to highlight the importance of blocking a single downstream target like TEAD in order to inhibit cancer progression.

**Figure 2:**
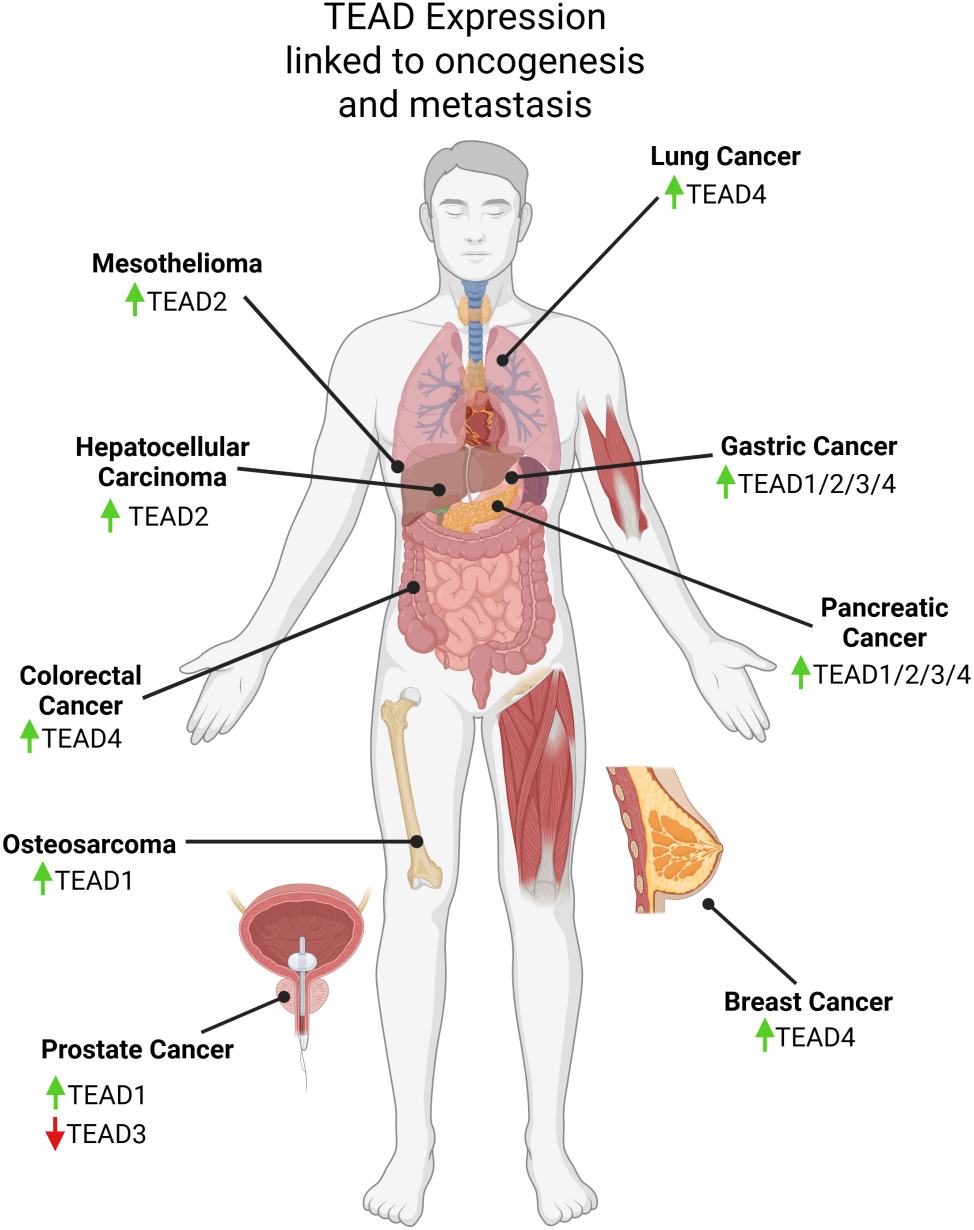
Graphical visualization of expression changes in TEAD family members linked to oncogenesis, metastasis, and patient survival discussed in this review. Created in BioRender. Battina, R. (2025) https://BioRender.com/1kkcq8x.

As noted above, the oncogenic activity of YAP/TAZ depends largely on its direct binding with TEAD, therefore, it is important to consider how the TEAD family itself contributes to tumorigenesis. Overexpression and hyperactivation of TEAD has been associated with tumorigenesis and cancer progression in a number of cancer types (**Figure 2**) (39). Similar to YAP/TAZ, high levels of TEAD in solid tumors is correlated with worse clinical outcomes (74–78). High mRNA expression levels of TEAD4 in approximately 4,000 breast cancer patients were correlated with a worse OS (79). Likewise, high nuclear protein levels of TEADs in tissues from lung adenocarcinoma and PDAC patients was also linked to a shorter OS (80, 81). Studies using mouse models have revealed the importance of TEAD in tumorigenesis. For example, TEAD4 knockdown in HCT116, a colorectal cancer cell line, suppressed proliferation and tumor growth in BALB/c mice (82). A TEAD2 dominant negative construct was used to inhibit YAP activity in MSTO-211H cells, a mesothelioma line, by competing with endogenous TEAD leading to loss of proliferation *in vitro*, and tumor regression *in vivo (*83). YAP1 shRNA knockdown and pharmacological inhibition using TEAD inhibitor K-975 in MSTO-211H cells engrafted in a mouse model prevented tumor initiation and induced tumor regression (83). Another group developed a truncated variant TEAD2 lacking a DNA-binding domain to inactivate TEAD activity in the liver (84). Surprisingly, this did not affect normal liver growth, but did curb hepatomegaly typically induced by YAP overexpression. In head and neck squamous cell carcinoma (HNSCC), use of SW-682 to block YAP/TAZ-TEAD binding led to an antitumor response in FAT1-mutant HNSCC xenograft models (85). TEAD inhibitor, NSC682769, was found to inhibit proliferation and migration while enhancing anti-tumor response in a glioblastoma xenograft mouse model (86). Another study demonstrated that gastric cancer proliferation is linked to PRIM1 expression which is transcriptionally regulated by TEAD4 (87). Through experiments using TEAD inhibitors (mechanisms of TEAD inhibitors are discussed in section 6), VT101, VT102, and VT103, it has been shown that targeting the Hippo pathway is a potential therapeutic strategy to block cell proliferation in NF2-null primary schwannoma and meningioma cells from human tumors (36). Periostin-CRE;NF2^fl/fl^ mice were treated with VT1 and VT2 leading to a shrinkage of schwannoma tumors *in vivo* (36).

However, it is important to note that YAP and TAZ can act as tumor suppressors for certain cancers and thus disrupting their interaction with TEAD will not necessarily be effective in all cancer types or at all stages of cancer. In androgen receptor positive (AR^+^) prostate cancer, YAP impeded tumor growth by competing with AR for binding and TEAD-mediated signaling (88). TEAD3 also inhibited proliferation and metastasis in prostate cancer (89). In primary small cell lung cancer (SCLC), IHC revealed low levels of YAP/TAZ and gain-of-function studies demonstrated that increasing YAP reduced tumor burden and increased survival (90). In renal clear cell carcinoma, YAP has been shown to repress proliferation that was driven by a TEAD-NFκB transcriptional complex by competing for binding to TEAD (91). In ER^+^ breast cancer, high levels of YAP lead to a good prognosis and inhibit ERa/TEAD interactions suggesting that YAP competes with ERa to block cancer growth (92).

Hippo pathway signaling has been shown to greatly impact cancer progression which is why it’s important to understand structural components of TEAD to develop inhibitors that can alter transcriptional regulation as an avenue for therapy.

## 5 Structure and homology of TEAD

**Figure 3:**
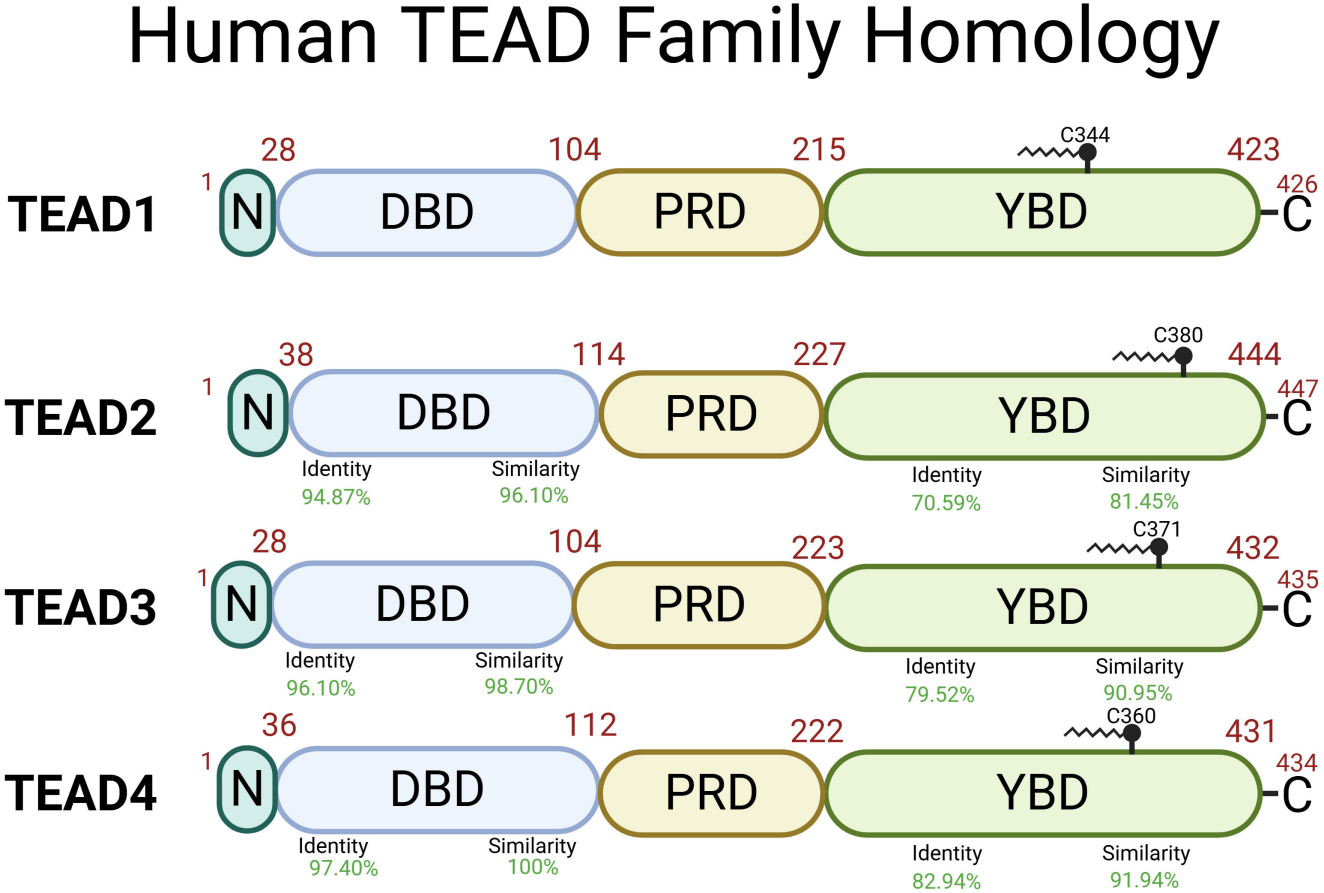
Human TEAD1–4 proteins share two highly conserved regions with the DNA Binding Domain (DBD) and the YAP Binding Domain (YBD) and less conserved regions including N-terminal domain (N) and the proline-rich domain (PRD). TEAD1 (Uniprot ID: P28347), TEAD2 (Uniprot ID: Q15562), TEAD3 (Uniprot ID: Q99594), TEAD4 (Uniprot ID: Q15561) structural function is dependent on palmitoylation at Cys344, Cys380, Cys371, and Cys360, respectively (93). Identity (DNA sequence) and similarity (amino acid sequence) percentage are relative to TEAD1. Created in BioRender. Battina, R. (2025) https://BioRender.com/8wzltok.

Within the TEA domain, TEAD1–4 have greater than 95% amino acid similarity (**Figure 3**) (94). Initial studies used nuclear magnetic resonance spectroscopy to propose a model for the protein structure (95). The DNA binding domain (DBD) also known as the TEA domain consists of three alpha-helices forming a globular structure. It was discovered that the L1 loop was essential for TEAD loading to M-CAT sites and helix H3 was the DNA-recognition helix of TEAD (95).

**Figure 4:**
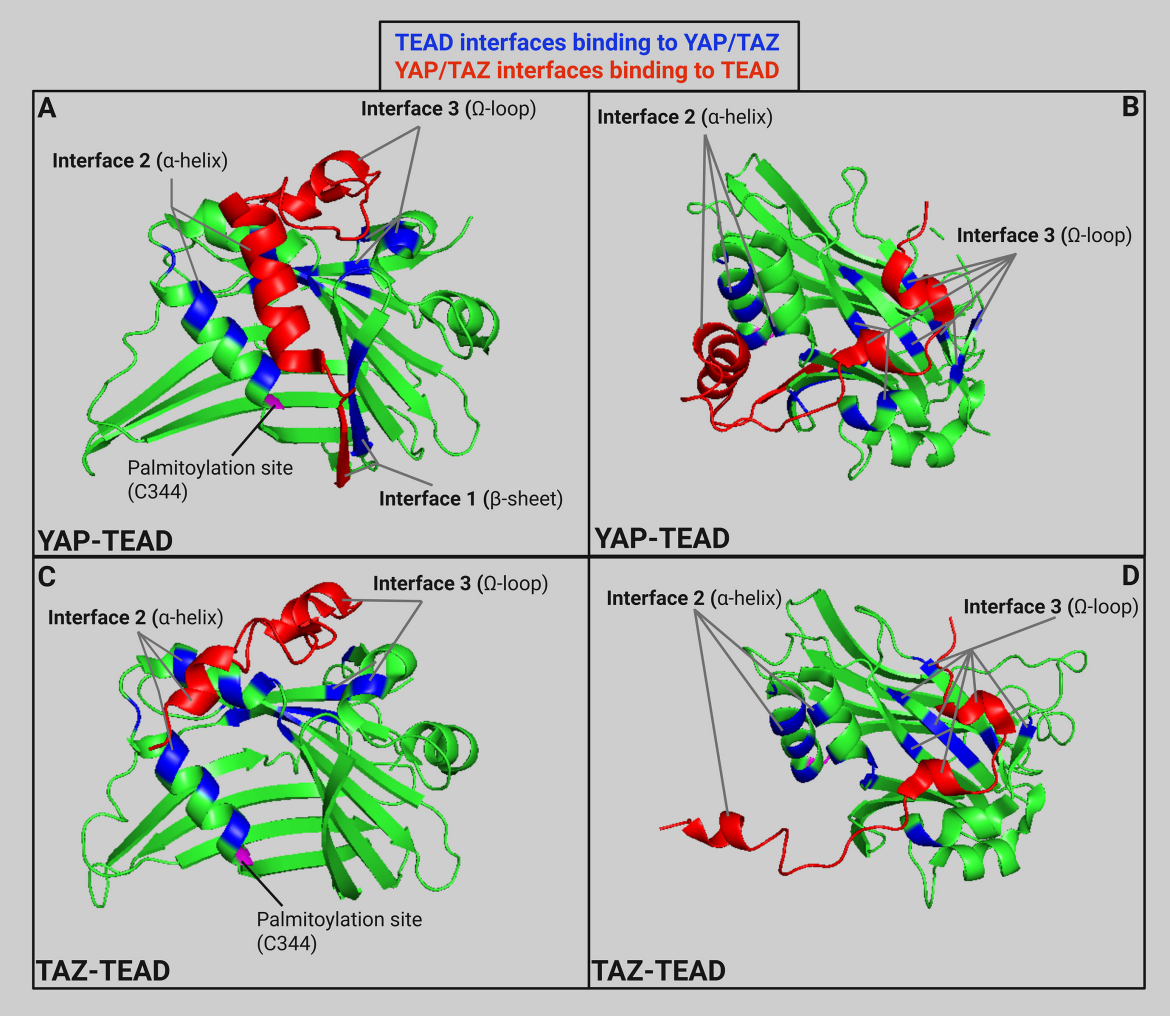
**(A)** Ribbon model (PDB: 3KYS (96)) of TEAD1 (amino acids 191-411) in green with specific TEAD interfaces in blue and partial YAP structure (amino acids 52-100) in red to highlight three main interfaces for YAP-TEAD binding. **(B)** YAP-TEAD interface rotated 60° on the x-axis and -60° on the z-axis. **(C)** Ribbon model (PDB: 5GN0 (97)) of TEAD1 (amino acids 191-411) in green with specific TEAD interfaces in blue and partial TAZ structure (amino acids 26-57) in red to highlight the main interfaces for TAZ-TEAD binding. **(D)** TAZ-TEAD interface rotated rotated 60° on the x-axis and -60° on the z-axis. Ribbon models created in Pymol and labeled using Biorender (98). Created in BioRender. Battina, R. (2025) https://BioRender.com/6ltz8nc.

The other highly conserved domain in TEAD is the YAP binding domain (YBD) which is in the C-terminal region. X-ray crystallography shows YAP-TEAD1 binding in residues 194–411 forming three highly conserved protein-protein interfaces (**Figure 4**) (96). The first interface (β-sheet) is mediated by seven hydrogen bonds between YAP β1 (residues 52-58) and TEAD1 β7 (residues 318-324) which forms an antiparallel β-sheet. The second interface (α-helix) is mainly mediated by hydrophobic interactions between the YAP α1 helix (residues 61–73) and a binding groove formed by TEAD1 α3 and α4 (residues 345-369). The final interface (Ω-loop) is between the twisted-coil region of YAP and a deep pocket in TEAD1 formed by β4, β11, β12, α1, and α4. This Ω-loop was also found when examining YAP-TEAD2 binding indicating that it plays a major role (99). Experiments repeated in mice examining YAP-TEAD4 binding also found three interfaces of binding (100). The β-sheet interface is between YAP (residues 65-70) and the TEAD4 β6-β7 loop. The α-helix interface is between the α1 helix and a binding groove formed by TEAD4 α3 and α4. The Ω-loop interface is between the α2 helix and strands β4, β11, and β12 of TEAD4. The YAP binding domain sequence of TEAD2 is 79-91% similar in TEAD1, 3, and 4 (101). There is a high level of similarity between binding of YAP and TEAD across species and family members indicating high conservation of YAP-TEAD interactions due to conservation in YAP-binding domain of TEAD and TEAD-binding domain in YAP (102). However, the hydrophobic proline-rich domain (PRD) and the N-terminal regions of TEAD are not as highly conserved across family members and different species. Initially, these domains were demonstrated to play a role in protein-protein binding between TEF-1 and scalloped in Drosophila (103). Another study found that deletion of the proline-rich domain within amino acids 115 to 223 cut YAP-TEAD binding to 57% efficiency (101). However, all deletions within the YBD showed an even stronger effect on binding efficiency, which ranged from 1-18%, highlighting the importance of interactions between YAP and the YBD. Subsequent crystallography experiments further identified those key interfaces within the YBD shown in **Figure 4**, but evidence pertaining to the exact role of the PRD was not discovered during these crystallography experiments. We suggest that the PRD may help enhance binding between YAP and TEAD, but does not play a major role which is why development of targeted therapeutics should be aimed against disrupting interactions between the interfaces of the YBD and TEAD.

While YAP and TAZ both bind to similar binding sites on TEAD, small alterations in protein structure affect the interactions between the proteins (**Figure 4**). For example, the major interface between the twisted-coil of YAP and the TEAD binding pocket is altered in TAZ due to a shorter loop (13 residues in TAZ versus 19 residues in YAP) (97). This leads to two alternate binding conformations between TAZ and TEAD. The first binding conformation is similar to the YAP-TEAD binding previously discussed. However, the main difference is the conformation of the smaller loop in the TEAD binding pocket. This second conformation is a heterotetramer conformation between two TAZ and TEAD molecules where TAZ helix α1 binds to the first TEAD while TAZ helix α2 binds to the second TEAD. The differential binding of YAP and TAZ to TEAD offers the opportunity to develop inhibitors that selectively disrupt either YAP-TEAD or TAZ-TEAD interactions without affecting the other complex. However, as we discussed previously, both YAP and TAZ have a role in many cancer types progression. Further studies are needed to indicate the benefit of developing selective inhibitors of these paralogs to block their differential effects on cancer progression.

The vestigial-like (VGLL) family of coactivators consists of four members, VGLL1-4 (see section 7) that interact with TEAD. Structurally, VGLL1 interacts with TEAD4 through two different interfaces. The first interface is an antiparallel β sheet between β2 of VGLL1 and β7 of TEAD4 and the second interface is between an alpha helix in VGLL1 and TEAD helices α3 and α4 (104). These interfaces, also known as the TONDU domain, are well conserved among the VGLL family (105). Despite the lack of primary sequence similarity between VGLL1 and YAP/TAZ, these proteins interact with the same two interfaces on TEAD though a VGLL1 analog to the Ω-loop interface was not found. However, further investigation by another group found that VGLL2 does have the Ω-loop interface unlike VGLL1/3 (106). VGLL4 is unique among the VGLL family as it has a secondary TONDU domain (104, 107). Interestingly, if this secondary TONDU domain is deleted, it prevents VGLL4 from inhibiting lung cancer cell growth by attenuating YAP/TAZ activity (108). As we will discuss in section 7, this leads to VGLL proteins having highly context-dependent effects on cancer progression.

Furthermore, it was found that TEAD undergoes autopalmitoylation at conserved cysteine residues (see **Figure 4**). One study mutated the conserved cysteine residue to an alanine in human TEAD2 which decreased overall protein levels and demonstrated that palmitoylation is important for protein stability (93). Another study identified that loss of TEAD palmitoylation significantly reduced co-immunoprecipitation between TEAD and YAP/TAZ, but was dispensable for binding to VGLL4 (109). Even after translation, TEAD proteins have been shown to undergo phase separation and form condensates intrinsically which serve to spatially regulate YAP/TAZ signalling (110). These structural discoveries have helped immensely in the development of TEAD inhibitors to prevent binding between TEAD and YAP/TAZ cofactors without potentially impacting other TEAD binding partners.

## 6 Key downstream targets of TEAD signaling in cancer

TEAD activation is implicated in tumorigenesis and cancer progression by promoting the transcription of genes involved in EMT, proliferation, cancer stem like (CSC) properties, drug resistance, and metastasis (39). EMT is a well-established process necessary for normal development, but is also exploited in cancers to facilitate tumorigenesis and metastasis (111). TEAD activation upregulates the expression of the canonical EMT markers Slug, ZEB1, ZEB2, and Vimentin (42, 77, 112–114). This in turn promotes the expression of mesenchymal genes, driving tumor cell invasion and migration. *In vitro* studies disrupting TEAD activity have further revealed this relationship. In ovarian cancer cells, SKOV-3, pharmacological disruption of the YAP/TAZ-TEAD complex resulted in a decrease in vimentin expression (115). Additionally, in a constitutively active TAZ^4SA^-mutant cell line, vimentin and N-cadherin were upregulated while epithelial markers E-cadherin and occludin were downregulated (116). Collectively, this data indicates the role of TEAD in regulating the gene expression of EMT markers (115).

Two well-known downstream targets of TEAD are CTGF and CYR61, both of which are part of the tissue growth factor family and function to regulate cellular proliferation, migration and adhesion (117). They have been implicated in multiple cancers as promoters of carcinogenesis and metastasis (118–123). CTGF has been shown to be upregulated in cancers of the brain, esophageal, breast, pancreas, melanoma and prostate (124–128). YAP/TAZ/TEAD binding leads to the transcription of CTGF, which promotes the activation of the FAK/Src/NFκB p65 signaling axis leading to increased cellular proliferation and migration through Glut3-mediated expression (129). CTGF has also been linked to chemoresistance through upregulation of Bcl-xl and cIAP1 in breast cancer (130). Upregulation of CYR61 induces chemoresistance in triple-negative breast cancer by activating Wnt signaling, EMT and a CSC-like phenotype (131).

A well-known pathway that drives cancer progression is the PI3K/Akt pathway which has been shown to interact with YAP/TAZ-TEAD. YAP analog, Yki, was found to require insulin-like growth factor (IGF-1) signaling through PI3K in order to translocate to the cytoplasm in Drosophila while inactivation of Akt prevented nuclear localisation of Yki (132). This PI3K-mediated activation of downstream Hippo signaling is reciprocated by YAP which was found to inhibit translation of tumor suppressor, PTEN, through miR-29 and overexpression of YAP in transgenic mice was shown to have diminished PTEN expression in IHC staining (133). A systematic gain-of-function kinase screen identified PI3KCB to positively promote YAP/TAZ function *in vitro (*134). A combination of PI3KCB and TAZ coexpression also led to tumor formation in mice despite using nontumorigenic human mammary cell line MCF10A (134).

TEAD is also involved in regulation of MYC, one of the most commonly activated oncoproteins in cancer (135). It has been found that overexpression of MYC leads to a partial rescue of cellular proliferation following YAP/TAZ depletion (136). Based on this, MYC is not only a downstream regulator of oncogenesis affected by TEAD activation, but can also play an important role in YAP/TAZ-TEAD signaling leading to cancer progression. A recent study has found that mesothelioma cells resistant to the TEAD inhibitor, K-975, exhibited increased activation of MYC signaling and continued to proliferate in a TEAD independent manner (137). This indicates that MYC activation can bypass the pharmacological inhibition of YAP/TAZ-TEAD signaling in cancers where Hippo pathway is mutationally inactivated. Interestingly, other studies have found that upregulation of MYC can repress YAP/TAZ activity in triple-negative breast cancer, indicating a need for further research to better understand these interactions (138, 139).

## 7 Mechanisms of action for TEAD inhibitors

**Figure 5:**
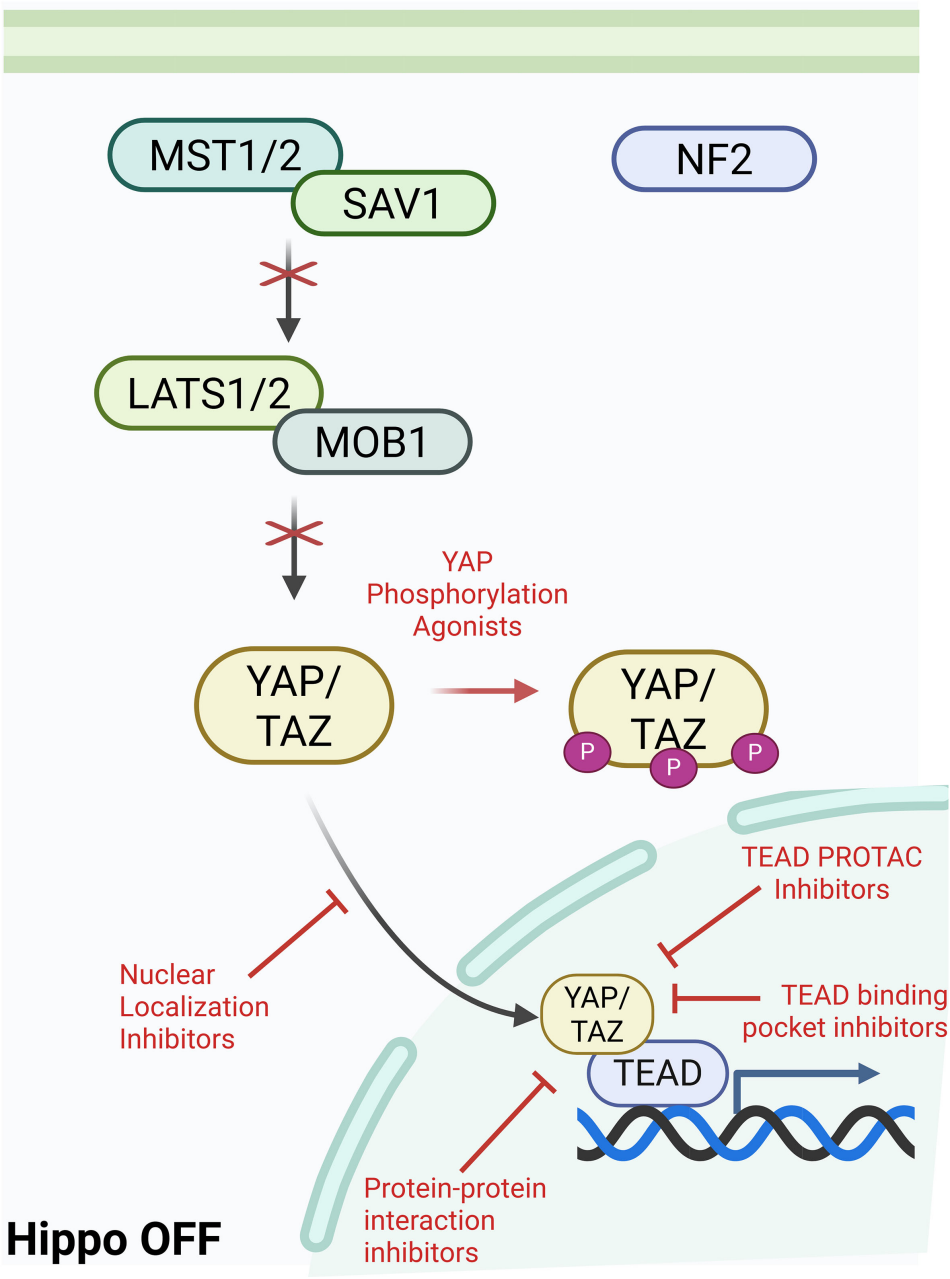
Mechanisms of Action for TEAD inhibitors currently in development or clinical trials. Created in BioRender. Battina, R. (2025) https://BioRender.com/of9ygmt.

The correlation between the dysregulation of the Hippo pathway and oncogenesis has been identified in multiple cancers so multiple pharmacological agents have been developed to target the interactions between YAP and TAZ with TEAD and reduce further downstream activation of the related oncogenic genes. A few different mechanisms of action have been developed to adequately target this complex, including YAP phosphorylation agonists, YAP nuclear localization inhibitors, protein-protein interaction inhibitors, and TEAD binding pocket inhibitors (**Figure 5**).

(i) YAP phosphorylation agonists increase degradation of YAP/TAZ, preventing localization into the nucleus and binding to TEAD. A35 is a topoisomerase inhibitor that was able to decrease YAP nuclear localization and inhibit growth in human leukemia cells by increasing phosphorylation of YAP (140). Furthermore, dasatinib and fluvastatin changed actin dynamics and induced YAP/TAZ phosphorylation in breast cancer cell line, MDA-MB-231 (141). MDA-MB-231 cells treated with dasatinib or fluvastatin also demonstrated increased sensitivity to doxorubicin and paclitaxel treatment.

(ii) YAP nuclear localization inhibitors can prevent or even reverse the nuclear translocation of YAP. Statins such as cerivastatin and simavastatin have been shown to inhibit nuclear localization of YAP through re-localization of YAP from the nucleus to the cytoplasm (142). Pazopabin plays a dual role as it can induce proteasomal degradation of YAP/TAZ and also induce phosphorylation of YAP/TAZ (141).

(iii) Protein-protein interaction (PPI) inhibitors block the YAP/TAZ interface interactions with TEAD described previously. IAG933 is a pan-TEAD PPI inhibitor. As a competitive antagonist, it directly prevents the formation of a transcriptional complex between YAP/TAZ and TEAD by blocking protein-protein interactions at the third interface binding site (143). Another successful PPI inhibitor is Super-TDU which is a peptide that mimics VGLL4 and orthosterically competes with YAP/TAZ for binding to TEAD. In a gastric carcinogenesis murine model, Super-TDU reduced tumor volume and mRNA levels of CTGF and CYR61 (144). A TEAD dominant-negative protein inhibitor (TEADiv2) structurally based on the YBD strongly inhibited transcriptional activity of TEAD in HEK293 cells by 5-fold (145).

While not a direct PPI inhibitor, GNE-7883 has been shown to block TEAD allosterically at the lipid binding pocket causing conformational changes that lead to tension between the two TEAD helices that compose part of interface 2 (see **Figure 4**) while also displacing a proximal residue, Gln410 (146). GNE-7833 reduced growth in YAP/TAZ dependent or NF2-null cell lines including OVCAR-8, MDA-MB-231, NCI-H226, and HCC1576 (146). Furthermore, addition of GNE-7833 overcame resistance to sotorasib in NSCLC in mice.

(iv) TEAD auto-palmitoylation inhibitors. Recently, it was discovered that the TEAD family is palmitoylated at a universally conserved cysteine allowing for proper TEAD folding and stability (93). Multiple studies found that disrupting palmitoylation of TEAD affects protein stability and function and can also prevent binding to chromatin which would lead to reduced transcription in both cases (93, 147). This led to development of several small molecule TEAD binding pocket inhibitors that block palmitoylation to impair TEAD function. K-975 covalently binds to the internal cysteine in the palmitate-binding pocket and suppresses proliferation in NF2-deficient mesothelioma cell lines and xenograft models (148). MGH-CP1 blocks autopalmitoylation of TEAD and showed significant overlap in gene expression changes with YAP/TAZ siRNA in MDA-MB-231 cells. MGH-CP1 inhibited tumor initiation in Huh7 and MDA-MB-231 xenograft models. Furthermore, they found that MGH-CP1 led to AKT activation and combination with an AKT inhibitor led to cancer cell death (149). MYF-03–176 is a covalent inhibitor that binds to the key cysteine residue within TEAD to prevent palmitoylation and was shown to reduce proliferation in liposarcoma cell line, 94T778 (150). Another study was able to identify a small molecule inhibitor that dysregulated TEAD palmitoylation and caused TEAD to act as a transcriptional repressor and limited tumor growth in a Detroit X1–562 mouse xenograft model (147).

(v) TEAD PROTAC inhibitors. Another mechanism developed for targeting TEAD is the use of proteolysis targeting chimeras (PROTAC) to promote degradation of the target protein through binding to E3 ubiquitin ligase. HC278, a TEAD-specific PROTAC, is able to degrade TEAD1/3 at nanomolar doses (151). Further testing in HC226 mesothelioma cell lines also showed a reduction in colony formation. Another set of PROTACs was developed by linking a VT107 analog to a thalidomide ligand and optimized by evaluating the antiproliferative effects against NF2-deficient HC226 cells (152). They found that compound 27 led to selective TEAD2 degradation and reduced transcription of YAP target genes. One paper identified TEAD PROTAC, H122, that induced TEAD1 degradation at nanomolar concentrations and had antitumor response in a MSTO-211H mouse xenograft model (153). Another PROTAC, Compound D, demonstrated significant degradation of TEAD1/3 in MDA-MB-231 and OVCAR-8 cells (154). Compound D also reduced accessibility to chromatin at TEAD motifs and inhibited cell proliferation in OVCAR-8 cells. Furthermore, there are patents out for more TEAD PROTACS (WO/2021/178339 and WO/2023/031801) as well indicating that this is a rapidly growing class of TEAD inhibitors and we expect to see more preclinical and clinical data regarding their use in cancer.

Some of these TEAD inhibitors have reached clinical trials and are currently being assessed for their effectiveness in metastatic malignant mesothelioma and other solid tumors (**Table 1**). Currently, there are several TEAD inhibitors in human clinical trials including VT3989 (NCT04665206), IAG933 (NCT04857372), ISM6331 (NCT06566079), SW-682 (NCT06251310), ION537 (NCT04659096), and BPI-460372 (NCT05789602). A previous clinical trial (NCT05228015) and further research on TEAD1-selective inhibitor, IK-930, was discontinued based on a review of the clinical data. As TEAD inhibitors are a newly developing drug class, all current clinical trials are still in early phase trials and many have not publicly reported their clinical findings at this time.

**Table 1:** TEAD inhibitors being investigated in cancer clinical trials.

Drug name	Developed by	Clinical trial #	Phase	Mechanism of action	Reported adverse events	Reference
IAG933	Novartis Pharmaceuticals	NCT04857372	Phase 1	Protein-Protein Interaction Inhibitor	Preclinical albuminuria and renal tubular injury	(143, 155, 156)
VT3989	Vivace Therapeutics	NCT04665206	Phase 1/2	Auto-palmitoylation inhibitor	Clinical proteinuria, albuminuria, peripheral edema, fatigue, and nausea	(157–159)
ISM6331	InSilico Medicine	NCT06566079	Phase 1	Auto-palmitoylation inhibitor	Safety profile not reported	(160)
SW-682	SpringWorks Therapeutics	NCT06251310	Phase 1	Auto-palmitoylation inhibitor	No preclinical adverse events reported	(85, 161)
ION537	Ionis Pharmaceuticals	NCT04659096	Phase 1	YAP1 Antisense Oligonucleotide	Safety profile not reported	(162)
BPI-460372	Betta Pharmaceuticals	NCT05789602	Phase 1	Auto-palmitoylation inhibitor	No renal toxicity observed clinically	(163, 164)
IK-930	Ikena Oncology	NCT05228015	Phase 1 (Discontinued)	Auto-palmitoylation inhibitor	Clinical proteinuria, fatigue, nausea, and diarrhea	(165–167)
ODM-212	Orion Pharma	ISRCTN99739590	Phase 1	Not reported	Safety profile not reported	(168)
BGC-515	BridGene Biosciences	NCT06452160	Phase 1	Not reported	Safety profile not reported	(169)

### 7.1 VT3989

While the chemical structure of VT3989 has not been publicly disclosed, a previous study has shown preclinical efficacy of precursor small molecule inhibitors that also block TEAD auto-palmitoylation, VT101-VT107 (157). Using a YAP reporter assay, they were able to identify 153 compounds that reduced YAP/TAZ-TEAD based luciferase signaling by >80% at 5umol/L and led to the identification of VT101 and 102 which were able to downregulate expression of *CYR61* and *AMOTL2*. New analogs, VT103–107 were developed and VT104 and VT107, an enantiomer of VT104, were found to block palmitoylation of all four TEAD members. VT103 and VT104 also reduced proliferation of NF2-deficiency mesothelioma xenografts in mice and in multiple NF2-mutant or NF2-deficient cell lines. Further optimization led to the development of VT3989 (structure unavailable) which is currently being used in a phase 1/2 clinical trial indicating enhanced efficacy in comparison to the earlier compounds which had already demonstrated strong antitumor efficacy through loss of pan-TEAD activity (158).

VT3989 is a small molecule pan-TEAD inhibitor that works by inhibiting TEAD auto-palmitoylation (158). It has been shown to block proliferation of NF2-deficient mesothelioma *in vitro* and *in vivo (*159). Additionally, VT3989 enhanced effectiveness of EGFR inhibitors, MEK inhibitors, BRAF inhibitors, MET inhibitors, mTORC inhibitors and KRAS^G12C^ inhibitors compared to monotherapy alone (158). In the ongoing phase 1/2 trial, they have enrolled 172 patients for dose escalation (n = 85) and dose expansion (n = 87) including 135 with mesothelioma that have progressed on prior therapy regimens (158). The overall response rate (ORR) reported was 26% in 47 patients treated with clinically optimized dosing and ORR of 32% in 22 patients with mesothelioma when using clinically optimized doses and remaining within thresholds for urine albumin:creatinine ratio (UACR). They found that intermittent dosing (100mg QD, 2 weeks on, 2 weeks off) of VT3989 led to lower albuminuria (4.9%) when compared to continuous dosing (22.2%) (158). Some common treatment-emergent adverse effects from VT3989 included fatigue (40.1%), increased UACR (32.6%), proteinuria (28.5%), and nausea (28.5%) (158). Common treatment-related adverse events included increased UACR (31.4%), proteinuria (27.9%), fatigue (19.8%) and peripheral edema (23.3%) (158).Grade ¾ events were rare with increased UACR (1.7%) and dyspnea (1.2%) occurring in more than one patient. Examination of tumor biopsies from six patients also demonstrated that VT3989 was able to modulate expression of YAP-TEAD target genes including CYR61 and CTGF. Notably, they found that VT3989 was able to demonstrate antitumor activity regardless of NF2 mutation status. VT3989 has shown enhanced efficacy in preclinical combination studies and has encouraging initial results from the phase 1/2 study. We look forward to further clinical reports regarding the efficacy and safety of this drug once the trial is fully completed.

### 7.2 IAG933

IAG933 is a small molecule pan-TEAD inhibitor currently being investigated in a phase 1 clinical trial (NCT04857372) that directly blocks binding between YAP/TAZ and TEAD. Interestingly, IAG933 leads to an increase in VGLL4 repression (see section 7) of TEAD demonstrating the selective nature of the drug targeting YAP/TAZ-TEAD protein-protein interactions. In human xenograft mouse models, oral IAG933 treatment led to tumor regression at tolerable doses in mesothelioma (143). A comparison to VT104 and K-975 showed that IAG933 had a more potent effect on YAP/TAZ-TEAD target gene expression, but had a shorter half life in the blood and tumor than VT104 (143). Combination treatment with osimertinib, an EGFR inhibitor, also improved efficacy of osimertinib in NSCLC (143). It also showed promising results in combination with KRAS^G12C^ inhibitors in CRC and NSCLC tumors as well as BRAF inhibitor dabrafenib in BRAF^V600E^ mutated tumors (143). The preclinical data demonstrates selective inhibition of TEAD which is being further investigated therapeutically in solid tumors. It also demonstrates an alternative therapeutic use in conjunction with MAPK pathway inhibitors to halt cancer progression (143, 155). In preclinical studies, it was found that 60mg/kg QD led to elevated urine kidney markers such as albuminuria due to renal tubular injury and protein-losing nephropathy, but when this weekly dose was administered 3 days on/4 days off, there was a reduction in these biomarkers within the urine (156). Based on the efficacy in preclinical settings, we would expect IAG933 to have clinical potential, but the phase 1 results are still unreported.

### 7.3 ISM6331

ISM6331 was developed using *in silico* modeling followed by further optimization as a pan-TEAD inhibitor that blocks TEAD palmitoylation to disrupt YAP/TAZ-TEAD transcription and is currently being investigated in a phase 1 clinical trial (NCT06566079) (160). It demonstrated strong antitumor efficacy in NF2-deficient mesothelioma models as monotherapy (160). Additionally, like IAG933 and VT3989, it showed synergistic effects with EGFR and KRAS^G12C^ inhibitors (160). However, there are no preclinical or clinical reports on the safety of this drug to date. Future reports on ISM6331 use in clinical trials could support further use of *in silico* modeling to develop novel TEAD inhibitors.

### 7.4 SW-682

SW-682 (NCT06251310) is a pan-TEAD small molecule inhibitor which blocks palmitoylation of TEAD to inhibit all TEAD-dependent transcription (161). It was reported that SW-682 inhibited proliferation of Hippo-mutant mesothelioma cells *in vitro* and tumor regression in murine mesothelioma models. Like other TEAD inhibitors, SW-682 synergized with EGFR and KRAS^G12C^ inhibitors for NSCLC PDX models *in vivo (*161). SW-682 was also shown to decrease cellular proliferation and downregulated YAP/TAZ-TEAD target genes in HNSCC cell lines Cal27 and Cal33 and in tumor xenograft models (85). It was also reported that SW-682 has good oral bioavailability in mice and did not lead to weight loss during treatment. However, there were no reports on the potential effects of SW-682 on renal function and urine biomarkers which have been reported with use of other TEAD inhibitors which could indicate a better side effect profile clinically.

### 7.5 ION537

Unlike other TEAD inhibitors discussed, ION537 is an antisense oligonucleotide that selectively targets YAP1 to cause a marked reduction in YAP1 protein level in HCC and HNSCC tumor models (162). Interestingly, ION537 treatment also led to an increase in T cell infiltration and enhanced efficacy of anti-PD1 antibody treatments. The results from the clinical trial (NCT04659096) have not been reported at this time. The unique nature of ION537 as an YAP1 antisense oligonucleotide coupled with an increase in immune infiltration indicate a novel mechanism of targeting TEAD which could also potentially sidestep the renal toxicity of other TEAD inhibitors.

### 7.6 BPI-460372

BPI-460372 is a small molecule inhibitor that covalently binds to the cysteine residue in the central binding pocket of TEAD thus irreversibly inhibiting palmitoylation. *In vivo* testing demonstrated reduction of tumor growth in NF2-deficient and LATS1/2-mutated xenograft models (163). Further studies found that dose escalation ranging from 10 to 80 mg per oral dose during the Phase 1 study (NCT05789602) did not lead to any Grade 3 or higher adverse events and did not observe any renal toxicity when administered using the 3 days on/4 days off schedule and improved renal injury biomarkers while still maintaining efficacy (164). Reducing the renal toxicity biomarkers while maintaining efficacy makes BPI-460372 a viable candidate should its safety and efficacy be validated clinically.

### 7.7 ODM-212

ODM-212 is a pan-TEAD inhibitor currently in Phase 1 (ISRCTN99739590) that is being evaluated for use in select advanced solid tumors (168). There is currently no published data regarding safety profile or preclinical results so future results from the Phase 1 trial will be critical for further consideration.

### 7.8 BGC-515

BGC-515 is a TEAD inhibitor currently in Phase 1 (NCT06452160) being evaluated for use in metastatic mesothelioma, epithelioid hemangioendothelioma, and other solid tumors. There is currently no published data regarding the safety profile of BGC-515. However, another TEAD inhibitor developed by BridGene is BGI-9004. BGI-9004 treatment led to tumor regression in xenograft models using mesothelioma cell lines, H226 and MST-0211H (169). They also did not see any deleterious effect on total body weight in mice during the 28 day treatment course. Dose response matrices suggest that BGI-9004 could be used in combination therapy with KRAS inhibitors in KRAS-mutant cell lines to overcome resistance (169). We hypothesize that BGC-515 may be the result of further optimization and preclinical testing following these discoveries with BGI-9004 and look forward to results from the Phase 1 trial.

### 7.9 IK-930

IK-930 is a small molecule inhibitor that selectively blocks autopalmitoylation of TEAD1 inhibiting proliferation of Hippo pathway-mutated cancer cell lines and antitumor effects in mesothelioma xenograft models (165). Furthermore, IK-930 enhanced the efficacy of EGFR and MEK inhibitors *in vivo*. It was reported that IK-930 drove interactions between VGLL4 and TEAD1 which repressed oncogenic activity and blocked chromatin association by other TEAD family members. Additionally, treatment with IK-930 had limited renal toxicity in rats and no renal issues in non-human primates (166). They reported that proteinuria was only recorded in 3 out of 26 patients and was fully reversible without dose-limiting effects (167). Other adverse effects reported were fatigue, nausea and diarrhea. Unfortunately, as the phase 1 clinical trial for IK-930 was discontinued, it is unlikely that there will be further investigation into its potential as a TEAD inhibitor despite the favorable renal safety profile and TEAD1 selective activity.

Overall, TEAD inhibitors are a very novel drug class that is still undergoing investigation, so there is a lack of extensive data publically available, however we anticipate that completion of these Phase 1 studies will provide greater insights on safety and efficacy of TEAD inhibitors clinically. Even with the limited data provided, a potential adverse effect generalized to TEAD inhibitors seems to be some form of renal toxicity leading to elevated urine biomarkers such as albuminuria. As discussed previously, different members of the TEAD family have tissue-specific expression patterns even as early as embryogenesis. High levels of YAP and TEAD3 and TEAD4 motif enrichment have been discovered to be important for podocytes differentiation and survival which could explain why some pan-TEAD inhibitors may have stronger adverse effects than paralog-specific inhibitors such as IK-930 which did not find renal toxicity in non-human primates and very little proteinuria in human patients (170–172). In all reports, it was indicated that this toxicity is reversible and has been shown to be mitigated through an altered dosing regimen in several drugs. This would still indicate that any synergistic treatment plans between TEAD inhibitors and other drugs that can increase renal toxicity should be carefully evaluated to ensure that dosing does not hinder patient survival and reduce long-term efficacy of treatment.

## 8 Alternate TEAD binding partners in cancer

YAP and TAZ are the most heavily studied binding partners for the transcription factor TEAD, but there are additional binding partners that enhance or suppress transcription through binding to the TEAD transcriptional complex.

### 8.1 AIB1/SRC3/NCOA3

Amplified in Breast Cancer 1 (AIB1) or the steroid receptor coactivator (SRC3) is an oncogene overexpressed in cancer that acts as a coactivator to enhance transcription through interactions with several transcription factors including TEAD. Early reports identified functional interactions between members of the SRC family and TEAD2 leading to hypotheses that SRC could be a coactivator for the TEAD family of transcription factors and bind directly to TEAD (173). In fact, knockdown of AIB1 in MCF10A reduced TEAD-mediated downstream expression of CTGF and ANKRD1 (174). Furthermore, knockdown of AIB1 also led to a reduction in TEAD-YAP luciferase reporter activity indicating its role to bridge interactions between TEAD and YAP. Further studies demonstrated that AIB1 physically interacts with YAP and TEAD in MCF10A and MCFDCIS breast cancer cells and that AIB1 can recruit the tumor suppressor ANCO1, a repressor of the S100 and SPRR gene families (175). ChIP-qPCR analysis showed that AIB1 was more enriched at ANKRD1 with TEAD and AP-1 occupancy than with a TEAD4 peak with only TEAD occupancy in HCT116 cells (176).

### 8.2 NFκB

While the interactions between NFκB and TEAD are not yet fully understood, it has been shown that TEAD1 can cause nuclear sequestration of p65 leading to development of a TEAD/p65 complex in rat pulmonary epithelial cell line, L2 (177). This complex regulates a subset of immune genes that were previously believed to be solely NFκB-dependent (177). Co-immunoprecipitation confirmed p65 formed complexes with TEAD1 and TEAD4. Interestingly, YAP was found to have a tumor suppressor role in clear cell renal carcinoma by competing with p65 for TEAD binding and excluding other protein interactions with TEAD (91). Furthermore, it was found that cytoplasmic YAP can directly attenuate NFκB activity by binding to TAK1 and IKK blocking downstream activation in primary chondrocytes (178). Further investigation of the binding between TEAD and NFκB in other cancers could potentially provide further insights on YAP’s paradoxical role as a tumor suppressor.

### 8.3 MEF2

Myocyte enhancer factor-2 (MEF2) transcription factor is involved in neural development, muscle formation, cardiac development, and is implicated in carcinogenesis (179). A co-immunoprecipitation assay in HeLa cells and cardiac myocytes demonstrated that TEAD1 and MEF2 physically interact and can interfere with MEF2-dependent activation of other promoters (180).

### 8.4 FAM181A/B

FAM181 A/B play a role in murine embryonic development and have also been found to interact with TEAD (181, 182). These proteins were identified from protein databases by looking for sequences mimicking the Ω‐loop region of YAP in other proteins. A co-immunoprecipitation experiment demonstrated that FAM181A/B were able to bind to TEAD4. However, they were unable to determine if FAM181A/B exerts an effect on TEAD transcriptional activity in HEK293FT cells. Like YAP/TAZ, FAM181A was blocked from TEAD binding after treatment with inhibitor IAG933 (110).

### 8.5 AP-1

Activator protein 1 (AP-1) is a transcriptional complex that is composed of multiple proteins including c-Fos, c-Jun, and ATF. *De novo* motif analysis of YAP/TAZ-TEAD peaks in MDA-MB-231 breast cancer cells found that AP-1 transcription factor was the next most frequent motif (136). A dominant-negative mutant of Jun repressed AP-1 signaling which consequently inhibited cellular proliferation and transcription of downstream TEAD genes. Another study performed ChIP-seq on A549, HCT116, and SK-N-SH cancer cell lines to find that TEAD4 and AP-1 co-occupied peaks were enriched for genes that regulate cellular migration and invasion (176). ChIP-qPCR analysis showed that SRC3 was more enriched at ANKRD1 with TEAD and AP-1 occupancy than with a TEAD4 peak with only TEAD occupancy (176). Using ChIP-seq in MDA-MB-231 cells, it was found that AP-1, STAT3, and TEAD co-localize at YAP/TAZ target sites suggesting that an AP-1/STAT3/TEAD complex recruits YAP/TAZ and increases expression of a AP-1 enriched motif gene subset that correlated with poor overall survival in triple-negative breast cancer (183). YAP and AP1 related transcription is activated during PDAC tumorigenesis and loss of AP-1 suppressed YAP-dependent cellular proliferation suggesting overlapping downstream targets (184). This was verified by co-precipitation of YAP, TEAD, and AP-1 in dysplastic lesions. TEAD and AP-1 motif enrichment have also been identified in ChIP-seq data from patient-derived colorectal cancer organoids (185). Interestingly, the YAP/TAZ-TEAD transcriptional complex induces FOS gene expression in HEK293 cells (186). Since Fos is a subunit in AP-1, this could increase presence of AP-1 to form a complex and increase transcription of proliferation related genes (186).

### 8.6 VGLL

The vestigial-like (VGLL) family of coactivators consists of four members, VGLL1-4. The interactions between VGLL and TEAD are highly context-dependent as coactivation has been shown to have both proliferative and anti-proliferative effects in cancer. VGLL4 competes with YAP for binding to TEAD which inhibits YAP/TAZ-TEAD target gene expression which has been shown to be oncogenic in multiple cancers (108). On the other hand, VGLL1 and VGLL3 have been shown to promote cancer cell proliferation through interactions with TEAD (104, 187). In endocrine therapy-resistance breast cancer, the VGLL1-TEAD complex induced EGFR expression and promoted growth of fulvestrant-resistance cancer cells (188). A VGLL2-NCOA2 fusion protein found in spindle cell rhabdomyosarcoma was found to strongly induce expression of TEAD downstream target genes (189). VGLL3 knockdown inhibited proliferation in myoblasts and soft-tissue sarcoma (18). VGLL4 gene expression is reduced in lung cancer and its increased expression suppresses proliferation of lung cancer cells by suppressing TEAD transcription (108). VGLL4 was also shown to enhance multiple TEAD inhibitors such as VT104, IK-930, GNE-7883, IAG933, and MYF-03–178 in removing YAP from the TEAD condensates while other members of the VGLL family did not produce a similar effect (110).

It is important to note that these binding partners do not bind to TEAD exactly like paralogs YAP/TAZ. It is possible that inhibition of YAP-TAZ/TEAD binding could even lead to greater binding to TEAD and an increase in specific downstream transcription. Further research is needed to fully investigate how these cofactors influence cancer progression in the presence or absence of YAP/TAZ-TEAD signaling.

## 9 Discussion

From upstream components such as LATS1/2 to the TEAD transcription factor and its co-activators, dysregulation in the Hippo pathway plays a critical role in tumorigenesis. TEAD has a key role in the Hippo pathway and can interact with multiple pathways, playing an important role in driving cancer progression which makes it an attractive therapeutic target. While the role of TEAD still needs to be fully elucidated, it’s clear that disrupting the interactions through the use of TEAD inhibitors is a promising clinical avenue.

TEAD inhibitors could play such a large role in the constantly evolving landscape of cancer therapy as there is a significant correlation between higher TEAD expression and decreased OS in numerous cancers such as breast cancer, lung cancer, and many others that were previously discussed. However, there is still more to learn regarding the role of Hippo pathway signaling in cancer. It has been identified that Hippo signaling can be protective in certain cancers suggesting that its downstream pathways can counteract oncogenic signaling driven by other commonly dysregulated pathways. The effects of TEAD inhibitors on stromal responses in cancer particularly the immune system is also an active area of investigation. We believe that this novel class of drugs should be further evaluated to understand the best practices for future treatment in patients expressing high levels of TEAD target gene transcription.

While no clinical trials have been completed, the safety and toxicity profiles of TEAD inhibitors are encouraging. Adverse effects such as nephrotoxicity were shown to be reversible with altered dosing schedules. Further information from early phase trials is needed before making any definitive statements on the safety profile of TEAD inhibitors, but there do not seem to be any major concerns. Despite the small number of patients, preliminary clinical findings from early phase trials indicate that TEAD inhibitors demonstrate activity against solid tumors so the promise of these drugs is recognized. This is particularly relevant because most current clinical trials focus on cancers with known mutations in Hippo pathway components, such as NF2; however, TEAD inhibitors may also suppress tumors independently of NF2 status. This suggests that blocking the oncogenic transcriptional activity of TEAD alone may be sufficient to limit tumor progression.

In conclusion, we believe that TEAD inhibitors have a promising potential and should continue to be pursued for further preclinical investigation and clinical studies.
